# Estimation of the mutation rate of *Mycobacterium tuberculosis* in cases with recurrent tuberculosis using whole genome sequencing

**DOI:** 10.1038/s41598-022-21144-0

**Published:** 2022-10-06

**Authors:** Jessica Comín, Alberto Cebollada, Aragonese Working Group on Molecular Epidemiology of Tuberculosis (EPIMOLA), María José Iglesias, Daniel Ibarz, Jesús Viñuelas, Luis Torres, Juan Sahagún, María Carmen Lafoz, Felipe Esteban de Juanas, María Carmen Malo, Sofía Samper

**Affiliations:** 1 Instituto Aragonés de Ciencias de la Salud, C/de San Juan Bosco, 13, 50009 Zaragoza, Spain; 2 Unidad de Biocomputación, Instituto Aragonés de Ciencias de la Salud, C/de San Juan Bosco, 13, 50009 Zaragoza, Spain; 3 Fundación IIS Aragón, C/de San Juan Bosco, 13, 50009 Zaragoza, Spain; 4 CIBER de Enfermedades Respiratorias, Av. Monforte de Lemos, 3-5. Pabellón 11, Planta 0, 28029 Madrid, Spain; 5 Universidad de Zaragoza, Zaragoza, Spain; 6 Hospital Universitario Miguel Servet, Zaragoza, Spain; 7 Hospital General Universitario San Jorge, Huesca, Spain; 8 Hospital Universitario Lozano Blesa, Zaragoza, Spain; 9 Salud Pública, Gobierno de Aragón, Zaragoza, Spain

**Keywords:** Bacterial genetics, Microbial genetics, Bacterial pathogenesis

## Abstract

The study of tuberculosis latency is problematic due to the difficulty of isolating the bacteria in the dormancy state. Despite this, several in vivo approaches have been taken to mimic the latency process. Our group has studied the evolution of the bacteria in 18 cases of recurrent tuberculosis. We found that HIV positive patients develop recurrent tuberculosis earlier, generally in the first two years (*p* value = 0.041). The genome of the 36 *Mycobacterium tuberculosis* paired isolates (first and relapsed isolates) showed that none of the SNPs found within each pair was observed more than once, indicating that they were not directly related to the recurrence process. Moreover, some IS*6110* movements were found in the paired isolates, indicating the presence of different clones within the patient. Finally, our results suggest that the mutation rate remains constant during all the period as no correlation was found between the number of SNPs and the time to relapse.

## Introduction

*Mycobacterium tuberculosis* has afflicted and co-evolved with man over thousands of years. Its success is due to its ability to infect a host and persist in a dormancy state for years^1,2^. During this period, the host is asymptomatic and not infectious, making the study of this state unmanageable. The bacteria stay in the granuloma, a barrier made by the immune systems cells, until not-well characterised signals or a weakening of the immune system allows the bacteria to escape and develop an active disease^3^. In recent years, some in vitro approaches have been designed for trying to increase the knowledge of latency^4^ and a debate about the physiological state of *M. tuberculosis* during this period has emerged. The number of mutations during a time period can be used as a molecular clock to study the evolution of the pathogen^6–9^. There were two studies with apparently contradictory results. Ford et al.^10^ used bacteria isolated from macaque lesions that mimic those of tuberculosis (TB) latent infection and concluded that generation time for latent TB would be similar to active TB, so the bacteria is physiologically active. On the other hand, Colangeli et al.^11^, studying an outbreak in New Zealand, concluded that generation times during latency are longer than during active TB. Recently Colangeli et al.^12^, pairing index cases to their TB contacts as a latency approach, concluded that both studies were correct: during the first two years of latency, the generation time is similar to that of the active disease, while later it starts to increase for long periods of time and a reduced mutation rate is observed. Looking for a novel in vivo approach, we carried out the analysis of isolates from individuals known to have developed several episodes of active TB for studying the evolution of the bacteria during the period between these episodes. Since 2004, all strains of *M. tuberculosis* have been genotyped in Aragon, Spain, which allowed us to identify the cases of TB relapses. The DNA previously used for genotyping remained in storage and could be used for whole genome sequencing (WGS) to analyse the variability of the isolates in the different episodes of the disease.

## Results

### Patient selection and risk factors

**Figure 1:**
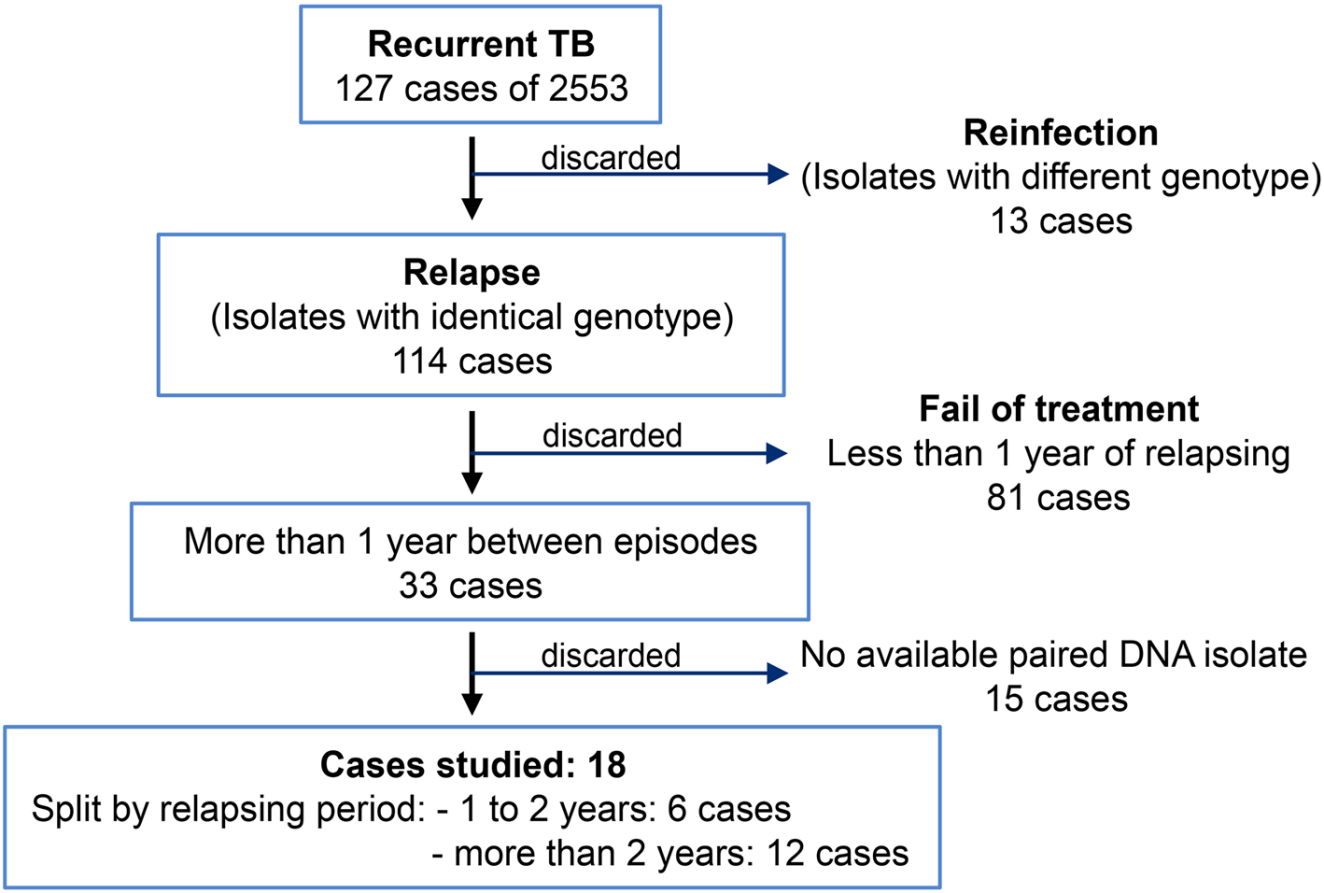
Diagram with the discarded and selected cases for the recurrent cases study.

**Figure 2:**
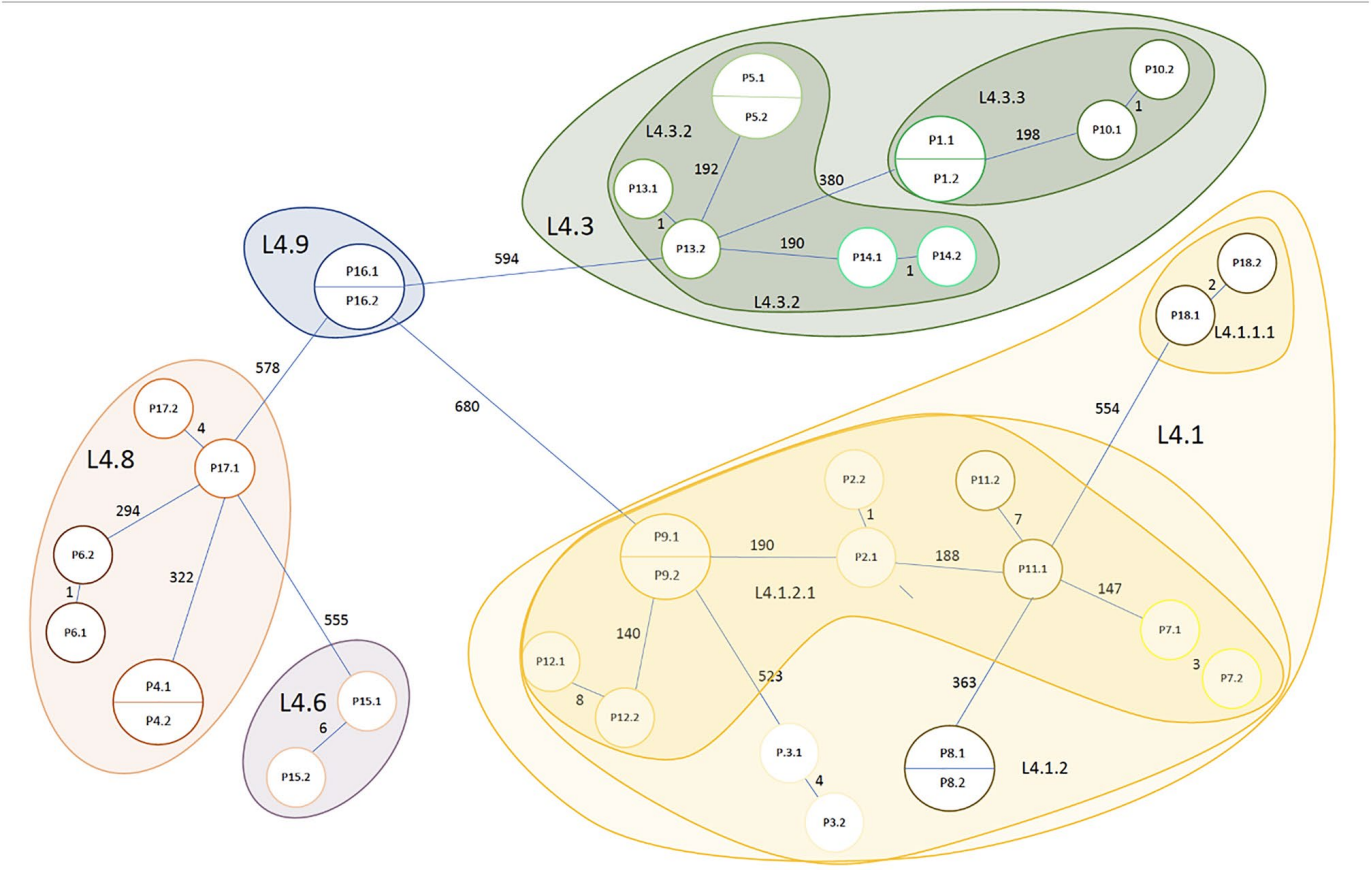
Drawing of a minimal spanning tree (not scaled) with all the studied isolates. The number of SNPs and the TB lineages are indicated.

The search of cases with recurrent TB among the total of cases in Aragon revealed 127 patients from 2004 until 2019 (4.97%). The genotype of the isolates revealed that 114 patients were infected by the same or very similar RFLP pattern strain, which would imply a potential relapse, while 13 patients were infected with non-related strains, i.e., re-infections, so these were not considered for this study. Among the potential relapses, we selected the cases with at least one year between episodes. Eighty-one patients had isolates with less than one year between them, therefore this group was discarded. Based on the time distance between the isolates, the cases were split into two groups: cases with ≥ 1 year but ≤ 2 years between isolates (12 patients), and cases with more than two years between the diagnosis of their isolates (21 patients). Eighteen pairs of *M. tuberculosis* isolates with available DNA of at least two different episodes were studied: twelve patients in the > 2 years group and six patients in the ≥ 1 but ≤ 2 years (Fig. 1). The lineages of the selected isolates are shown in Fig. 2.

Several selected patients had risk factors to develop TB. At least 38.9% were HIV+ , 22.2% declared a high alcohol consumption, 38.9% were smokers and 16.7% were intravenous (IV) drugs users. The treatment for all patients was the standard for susceptible TB. Despite some of them did not follow it correctly, no drug resistance was developed. We split the patients into two groups: [1–2 years until relapse] and (2–14 years until relapse] in order to investigate if some of these risk factors were related to a shorter or longer relapsing period (time between the first and the second episode). The intervals were fixed according to the results obtain by Colangeli et al.^12^, being 160 months the maximum time between episodes observed in our study. Results are shown in Table 1.

**Table 1:** Risk factors of the 18 selected cases.

	[1–2 years]	(2–14 years]	*p *value
	*N* = *6*	*N* = *12*	
**Gender**			0.316
Male	5 (83.3%)	6 (50.0%)	
Female	1 (16.7%)	6 (50.0%)	
**HIV status**			0.035
No	1 (16.7%)	8 (80.0%)	
Yes	5 (83.3%)	2 (20.0%)	
**Alcohol consumption**			1.000
High	1 (33.3%)	3 (37.5%)	
Low/No	2 (66.7%)	5 (62.5%)	
**Immunosuppression**			0.010
No	0 (0.00%)	7 (87.5%)	
Yes	4 (100%)	1 (12.5%)	
**Smoker**			0.491
No	2 (66.7%)	2 (25.0%)	
Yes	1 (33.3%)	6 (75.0%)	
**Users of IV drugs**			0.152
No	1 (33.3%)	7 (87.5%)	
Yes	2 (66.7%)	1 (12.5%)	

HIV status was significant (*p* value = 0.035) between the two groups, showing that HIV positive patients suffered relapse in the first two years more frequently than HIV negative patients.

### Analysis of the genomes

#### SNPs versus relapsing period

The number of existent SNPs between the first and its correspondent relapsed isolate ranged from 0 to 8. These SNPs were usually in the relapsed isolates, but we could also find some of them in the earliest isolates that then disappeared, showing different clones co-existing in the patient. The mutation effect of the SNPs and also the functional categories of the affected genes were analyzed; 26.3% belonged to cell wall and cell processes category and 21.1% to intermediary metabolism and respiration category. None of them were present in more than one case, therefore they do not seem to be directly implicated in relapsing. The detailed SNPs can be found in Table 2.

**Table 2:** SNPs found between the first and the relapsed paired isolates.

Pair	Relapsing period	N	Point	Gene	Functional category	Mutation effect
P1	13 m/8736 h	0				
P2	31 m/20,832 h	1	1,042,251	*pstS1*	Cell wall and cell processes	Non-synonymous
P3	92 m/61,842 h	4	2,793,634	*lipQ*	Intermediary metabolism and respiration	Non-synonymous
			3,031,623*	*Rv2719c:lexA*	Intergenic region	
			3,869,344	*Rv3448*	Cell wall and cell processes	Synonymous
			4,134,177*	*moxR2*	Regulatory proteins	Non-synonymous
P4	12 m/8064 h	0				
P5	51 m/34,272 h	0				
P6	13 m/8736 h	1	156,912	*fbpC*	Lipid metabolism	Non-synonymous
P7	64 m/43,008 h	3	1,480,972*	*Rv1319c*	Intermediary metabolism and respiration	Synonymous
			1,845,545	*uvrA*	Information pathways	Synonymous
			3,179,330	*Rv2867c:ispG*	Intergenic region	Synonymous
P8	160 m/107,520 h	0				
P9	31 m/20,832 h	0				
P10	31 m/20,832 h	1	245,322	*mmpL3*	Cell wall and cell processes	Non-synonymous
P11	42 m/28,224 h	7	144,506*	*fadD7*	Lipid metabolism	Non-synonymous
			157,655	*fbpC:htdZ*	Intergenic region	
			1,952,766*	*Rv1726*	Intermediary metabolism and respiration	Synonymous
			2,278,535	*hspX*	Virulence, detoxification, adaptation	Non-synonymous
			3,912,404	*mce4F*	Virulence, detoxification, adaptation	Non-synonymous
			456,028	*secE2*	Cell wall and cell processes	Non-synonymous
			1,837,204	*uvrB*	Information pathways	Non-synonymous
P12	39 m/26,208 h	8	1,049,482*	*Rv0939*	Intermediary metabolism and respiration	Synonymous
			2,124,862*	*Rv1875:Rv1876*	Intergenic Region	
			2,145,578	*lppD*	Cell wall and cell processes	Non-synonymous
			2,555,780*	*Rv2282c*	Regulatory proteins	Non-synonymous
			3,111,151*	*Rv2802c*	Conserved hypotheticals	Non-synonymous
			4,364,271*	*mycP1*	Intermediary metabolism and respiration	Non-synonymous
			4,394,960*	*Rv3909*	Conserved hypotheticals	Non-synonymous
P13	32 m/21,504 h	1	3,574,424	*Rv3201c*	Information pathways	Synonymous
P14	14 m/9408 h	1	1,543,706	*Rv1371*	Cell wall and cell processes	Synonymous
P15	104 m/69,888 h	6	1,505,180	*murI*	Cell wall and cell processes	Non-synonymous
			2,477,975	*Rv2212*	Intermediary metabolism and respiration	Synonymous
			2,675,882	*mbtB:mbtA*	Intergenic Region	
			2,701,688	*lepA*	Intermediary metabolism and respiration	Non-synonymous
			3,160,108	*Rv2851c*	Intermediary metabolism and respiration	Synonymous
			3,470,698*	*ftsE*	Cell wall and cell processes	Synonymous
P16	14 m/9408 h	0				
P17	59 m/39,648 h	4	1,840,457*	*Rv1634*	Cell wall and cell processes	Synonymous
			1,923,131	*Rv1698*	Cell wall and cell processes	Synonymous
			4,338,635	*whiB6:Rv3863*	Intergenic region	
			4,400,765	*sigM*	Information pathways	Non-synonymous
P18	16 m/10,752 h	2	397,372	*Rv0331:Rv0332*	Intergenic region	
			1,247,257	*ephC*	Virulence, detoxification, adaptation	Non-synonymous

Relapsing period (time between the first episode and the second) in months (m) and hours (h), Number of SNPs found between the paired isolates (N), Point referred to H37Rv genome, Gene name, Functional Category and the Effect of the SNP are detailed.

*SNPs detected in the first isolate and not in the relapsed isolate.

**Figure 3:**
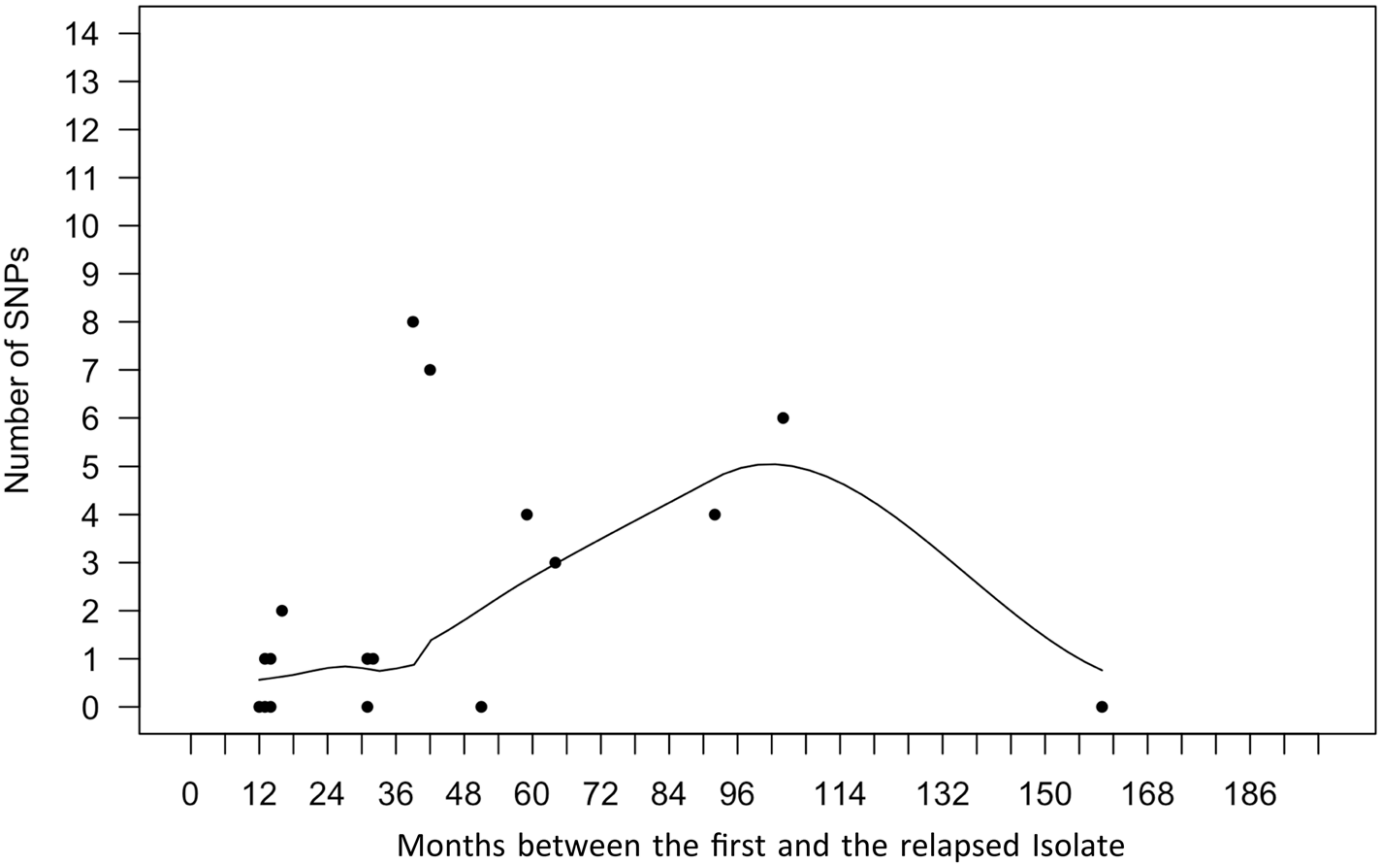
Scatter plot showing the number of SNPs developed between the first and the relapsed isolate of the patient versus time between the diagnosis of both isolates (in months). A Poisson regression model was used. A trend of increase in the number of SNPs is observed as the months of relapsing period increase, however, it was not significant (*p* value = 0.34).

In order to represent the number of SNPs developed between the first and the relapsed isolates of the same patient versus the time between the diagnosis of both isolates (in months), resembling in that way the latency period, we reproduced the study of Colangeli et al.^12^ using the Poisson regression model. Equally to Colageli et al. 2020, we found this correlation not significant (*p* value = 0.34), meaning that those isolates with a longer relapsing period were not necessarily those with more SNPs. The results are shown in Fig. 3. Otherwise, we observed that pairs of L4.1 had a higher mutation rate per genome per year (0.93 SNPs) than other sublineages (0.58 SNPs in L4.8 and 0.32 in L4.3), and it is above the average found by this study (0.64 SNPs).

#### Mutation rate versus generation time

**Figure 4:**
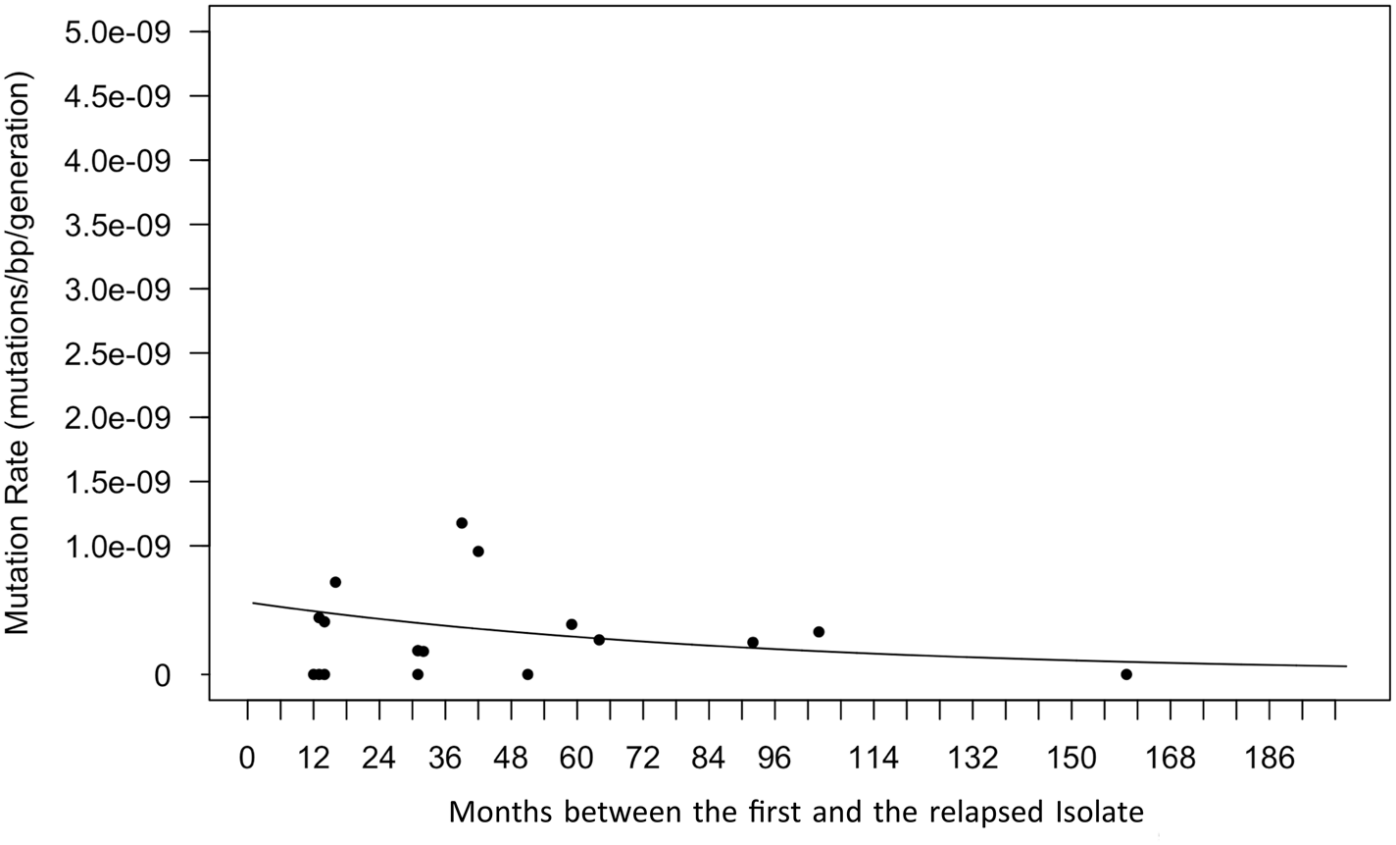
Mutation rate versus time until relapse. Scatter plot showing the number of SNPs that differed between the 18 patients’ paired isolates (y-axis) as a function of the time between episodes (x-axis). The generation time is held constant at 18 h as seen in actively replicating *M. tuberculosis* in vitro. The relation between the mutation rate and the time between episodes was marginally significantly different from 0.0 (*p *value = 0.0613), indicating a trend of a higher mutation rate in the first months of relapsing period.

In order to analyse the correlation between the mutation rate and the relapsing period, we used the Poisson model, as described by Colangeli et al. ^12^. The generation time was fixed at 18 h as seen in *M. tuberculosis* actively replicating in vitro. Results are shown in Fig. 4. The mutation rate tends to diminish in longer relapsing periods, being marginally significant (*p* value = 0.061).

**Figure 5:**
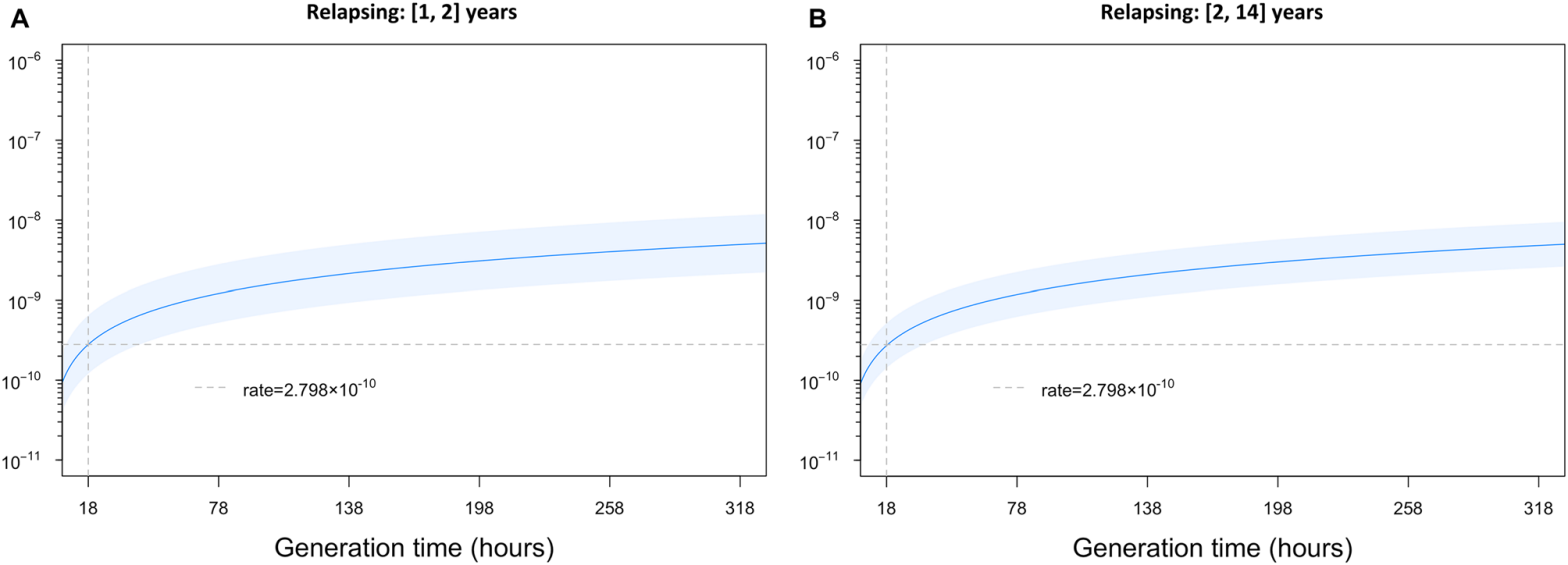
Changes in mutation rate during the relapsing period with varying generation times. Mutation rate (mutations per (bp × generation)) is shown for generation times ranging from 18 to 320 h for each pair grouped by the number of years between the first and relapsed isolates. The dark blue line is obtained from the regressions and shows the estimated mutation rate for a given generation time (x-axis) and light blue regions show 95% confidence intervals. The first panel shows the relationship between mutation rate and generation time during early relapsing (in ≤ 2 years) based on n = 6 pairs. The second panel shows the relation between mutation rate and generation time for relapse in 2–14 years based on n = 12 pairs. In both panels the grey dashed vertical line is fixed at 18 h, and the horizontal line indicates the mutation rate of 2.798 × 10^–10^ mutations per (bp × generation) as seen in early relapsing with generation times held constant at 18 h, fixed in both graphs for comparison availability.

When we considered the data in (1–2) and (2–14) years of relapsing periods, the mutation rate is slightly lower for the second, estimated at 2.728 × 10^–10^ [95% CI: 1.433 × 10^−10^, 5.193 × 10^−10^] mutations per (bp × generation), than in the first period, estimated at 2.798 × 10^–10^ [95% confidence interval (CI): 1.209 × 10^–10^, 6.477 × 10^−10^]. However, this difference was not statistically significant (*p* value = 0.96). Results are shown in Fig. 5.

#### IS6110 copies variation between the isolates

All the IS*6110* copies of the isolates were analyzed using the WGS data. We found several IS*6110* movements between the first and the relapsed isolates as a result of different clones. The relapsed strain gained extra IS*6110* copies in P7 (two copies gained) and P12 (one copy gained). On the other hand, we also observed four cases in which the relapsed isolate had lost some IS*6110* copies, present in the first isolate: P10 (one copy lost), P13 (three copies lost), P14 (one copy lost) and P15 (one copy lost). All these extra and absent copies usually had a lower number of reads than the fixed copies, indicating that they were not completely extended in the bacteria population. All these movements were observed in strains with more than 2 years between the isolates, except P14 pair (14 months). The IS*6110* locations can be found in Table S1.

## Discussion

Studying *M. tuberculosis* latency in humans is harsh due to the difficulty of isolating the dormant bacteria, which is not possible until the active disease. Much has been published regarding latent TB and the percentages of reactivation and disease, but the latency data in patients who have already passed the disease have not been studied. Different approaches were used to mimic this process^10–12^. This work shows, for the first time, results obtained using isolates of patients with recurrent TB. Aragon, a region in the North of Spain, has a low incidence of TB. Thanks to the surveillance protocol carried out in this region since 2004, all *M. tuberculosis* isolates are genotyped and registered, allowing to trace the clinical TB history of the patients. Around 5% of the TB cases in our population correspond to recurrent TB. Of them, 89.8% were TB cases with isolates showing identical IS*6110*-RFLP patterns, indicating a potential relapse. Most of them (71%) were later considered as fail of treatment. In contrast, 10.2% of the patients had isolates with different genotypes, considered as reinfections. Among the total of TB cases in our community, reinfection occurs in 0.5% of the TB cases, reflecting that reinfection is uncommon among our population. These data are in agreement with a previous study in Madrid population, which showed an 87.5% of relapses and 12.5% of reinfections among the cases with recurrent TB^13^. However, in a study in the Canary Islands, the results showed a higher reinfection percentage (44%) versus the 55% of relapses^14^. A more extreme result was obtained in a study in London, in which 72.6% of the repeated patients were classified as reinfections against a 27.4% of relapses^15^. The large variation of the results among the different studies suggests that they largely depend on the population sample studied. It would be very interesting to analyse the reinfection cases in each of the studies to understand the reasons for these differences. Regarding endemic TB regions, a higher percentage of recurrent TB was found. Around 9.5% of TB patients had recurrent TB in Malawi (39.6% had relapse and 14.4% reinfection, the rest was undetermined)^16^ and a study carried out in India demonstrated that the majority of relapses they had were among HIV negative people (95% of TB recurrences) while the majority of reinfections were among HIV positive people (75% of TB recurrences)^17^.

Regarding the epidemiological and risk factors of the relapsed TB cases studied, we found that relapse was significantly earlier in HIV positive patients (in the first two years since the first episode) when compared to HIV negative patients (*p* value = 0.035), what would be in accordance with a compromised immune system. Any risk factor was found as significant for causing an earlier reactivation by Colangeli et al. ^12^, however they recognized that the clinical cases studied did not have in general any comorbidity.

The number of SNPs between the pairs ranged from 0 to 8. Remarkably, three among the 18 pairs had more than 5 SNPs between the first and the relapsed isolate, interpreted as not recent transmission^18^, even though the bacteria were isolated from the same patient. This could be related to clinical characteristics of the patients, as immunosuppression, HIV status or the treatment adherence. Surprisingly, several SNPs were found in the first isolates that were absent in the relapsed isolates, as if they had reverted. This phenomenon was extreme in P12, in which six out of the seven SNPs found were absent in the relapsed isolate. The explanation could be the presence of different clones in the patient^19,20^*.* In this way, in the different disease episodes a different clone was isolated, resulting from different bottlenecks and selective pressures of the original strain^21,22^. The reinfection with an identical strain has been described as a limitation of these kind of studies, but in our case, it can be discarded as only one of the pairs belonged to a large endemic cluster (P4, with 0 SNPs). The rest of the pairs were infected with orphan or small-outbreak strains of up to four cases, differently from other studies with large endemic clusters and high TB prevalence^22^.

Same as Colangeli et at.^12^, we did not find a significant correlation between the number of SNPs and the time between episodes. However, it is possible that P8 (160 months between episodes and 0 SNPs) is altering the trend of SNP accumulation when the time between episodes increases. This is one limitation when working with small sample size, that a single point could have a great impact in the results. None of the SNPs found seemed related with recurrence as all were unique and therefore not common to more than one pair of isolates. It has been described that 0.5 SNPs per genome, per year is the standard mutation rate for *M. tuberculosis*^10^. Some studies, where multiple MDR/XDR isolates coming from the same patients were sequenced, have reported that selective pressure and antibiotic resistance can increase this mutation rate as high as more than 3 SNPs^17,21^. Despite all strains had been under the selective pressure of treatment, they did not achieve such a higher rate, maybe because they were drug susceptible. The mean mutation rate found in our study was 0.64 SNPs, slightly above the standard, due to the high mutation rate found in L4.1, almost double than the standard.

The correlation between the mutation rate and the relapsing period was found just marginally significant (*p* value = 0.0613), differently to Colangeli et al.^12^, who found it significant. It is important to remark that the approaches were completely different: they used transmission events to mimic the latency period as the time between the diagnosis of the two cases, while we used isolates from the same patient who had a previous TB episode. We eliminated all patients with less than one year between the diagnosis of the episodes, as this was considered as a treatment failure, while Colangeli et al. 2020 had latency periods from one month, which was not possible in our clinical cases as a minimum of 6 months of treatment was required. We did not find a significant correlation between the mutation rate along the variable generation times analysed when we split the data into [1–2 years] and (2–14 years), we observed just a small difference. This difference was much smaller than that found by Colangeli et al. 2020 (as high as 8 × 10^–10^ for early latency), suggesting that mutation rate was constant during the relapsing period in recurrent TB cases. The mutation rate found in our study, 2.7 × 10^–10^, was similar to that found by Ford et al. 2011 (2 × 10^–10^)^10^, therefore both more distant from the one found by Colangeli et al. 2020. The reason why our results are similar to those of Ford et al. 2011 could be due to the similarity of the approaches applied, as they used lesions of the same macaques for studying latency and we used relapsed isolates from the same patients.

The analysis of the IS*6110* element showed differences in the number of IS*6110* copies in six of the pairs studied, affecting more than one IS copy in several pairs. It has been observed that IS*6110* transposed more in great starvation conditions^23^, which could be similar to the conditions the mycobacteria found in the granuloma^4^. It was surprising that in four of the pairs studied, the relapsed isolates had lost 1 to 3 copies that were present in the first isolates. Noteworthy, the number of reads obtained in the fastQ files for these copies was considerably lower than for the rest of the IS copies. This suggests that those lost copies were not still fixed in the complete bacteria population, therefore a selection among the different clones present in the same patient had taken place^24^. It could be that the lost copies in the relapsed isolates had some deleterious effect for the mycobacteria as the relapsed bacteria were the ones without that IS copies. The fact that five out of the six pairs with IS*6110* movements had more than 2 years of relapsing period supports the idea of IS transposing more during the asymptomatic state of the patient^23^.

The main limitation to analyse the evolution of the bacteria during the dormancy period is the approach used for resembling this state. There is not a perfect approach, as it is impossible to reproduce what is happening inside the granuloma of a concrete patient, but we think that using isolates of the same patient is the closest way to do it. The difficulty to obtain the complete epidemiological information of the patients is another limitation because it does not allow to determine the accurate development of the disease’s episodes. Another limitation is that some of the SNPs could be the result of a sequencing error or due to laboratory management, what would have a huge impact on the mutation rate. In addition, although there were more cases of potential relapses in our records, DNA of the isolates was not available. We decided not to re-cultivate these stored isolates to avoid more manipulation that could introduce errors such as additional SNPs that were not present in the original strains.

As a conclusion, the patients with HIV seemed to suffer reactivation in the first two years after the initial episode of TB more frequently than HIV negative patients. Besides, IS*6110* movements occurred more frequently in patients with more than two years between episodes and it seems that different clones of the original strain could be responsible for the first and the following episodes. No correlation was found between the number of SNPs and the time between episodes, neither between the mutation rate and the relapsing period, just a trend of diminishing in longer time periods. Finally, the mutation rate seemed to be constant along all the period between episodes.

## Material and methods

### Selection of samples and patients

Of around 2553 cases of TB in Aragon since 2004, we first looked for those with more than one isolate more than 1 year apart and of a similar genotype. We used Bionumerics v6.7 software (v7.6, Applied Maths, Kortrijk, Belgium) to confirm that both isolates coming from the same patient shared an identical IS*6110*-RFLP pattern. Eighteen pairs of *M. tuberculosis* isolates with available DNA were included in this study. When there were more than two isolates from the same patient, the two more distant in isolation dates were considered for the evolution study during relapse. All data remained anonymous during the epidemiological search. Our regional ethical committee (Comité de Ética de la Investigación de la Comunidad Autónoma de Aragón, Record No. 20/2018) approved the methodology used in this work, detailed in 18/0336 project.

DNA of the bacterial isolates was obtained using the cetrimonium bromide method, as previously described^25^. No human DNA was sequenced. All DNA extractions were stored at – 20 °C until sequencing. All the isolates were genotyped by IS*6110*-RFLP and spoligotyping as previously described^26,27^. The genetic patterns obtained were stored and analysed in Bionumerics database software.

### SNP annotation and lineage identification

Thirty-six isolates corresponding to 18 different patients were sequenced using Ion Torrent technology according to manufacturer’s instructions. The fastQ files obtained were mapped against the reference *M. tuberculosis* strain H37Rv (NC_000962.3) in order to obtain the Binary Aligned Map (BAM) and Variant Call Format (VCF) files, used for the SNP study. The fastQ files were uploaded in Bionumerics software for the study and comparison of the genomes. The SNP annotation was carried out using Snippy software (default parameters) and Integrative Genomics Viewer (IGV), from the Broad Institute^28^. The effect of the mutation (synonymous or non-synonymous) was observed using Genewise platform (https://www.ebi.ac.uk/Tools/psa/genewise/). All the mutation points are referred to the H37Rv reference strain. For lineage identification, the SNP-based classification stablished by Coll et al. 2014^29^ was used. This classification assigns specific SNPs to each TB lineages and sublineages.

### IS*6110* location

All the reads containing the first and the last 30 base pairs of the IS*6110* sequence were extracted. These reads are formed by the beginning or the ending of the IS*6110* along with part of the gene in which the IS is inserted. After the extraction of the sequences, BLAST was made in Tuberculist and Bovilist to know the insertion point. BLAST was also made automatically with the script, but manual BLAST was required for some ambiguous points. The script used in R is in the Supplementary Materials.

### Mutation rate calculation

The mutation rate per (bp × generation) was calculated as previously described^10^, adjusting the parameters to our own data. Briefly, the mutation rate per bp × generation is defined as

$$\mu = \frac{{\mathop \sum \nolimits_{i = 1}^{n} m_{i} }}{{N\mathop \sum \nolimits_{i = 1}^{n} \left( {{{t_{i} } \mathord{\left/ {\vphantom {{t_{i} } g}} \right. \kern-\nulldelimiterspace} g}} \right)}}$$

where μ is the mutation rate, m is the number of SNPs between the first and the relapsed isolate, N is the genome size (since we had, on average, reads covering 97.4% of the *M. tuberculosis* genome, N = 0.974 × L where L is reference genome size), t is time since infection (in hours, Table 1), and g is generation time (in hours).

### Statistical methods

Poisson regression was used to model the variation of mutation rate over a range of generation times. To control the deviations from distributional assumptions a robust variance of Robust Sandwich Estimator was used. Poisson models were used to obtain mutation rates per (bp × generation) by using bp × generation as an offset. Two Poisson models were fit according to the relapsing period (one for 1–2 years including n = 6 pairs and another model for 2–14 years including n = 12 pairs). We also fitted a Poisson model using the relapsing period as a continuous independent variable. The hypothesis that we tested was if the parameter associated to relapsing period was significantly different from 0. To test the Poisson model parameters, a two-sided chi square test using the robust variance was used. Software R version 4.0.5 (2021-03-31) was used to all statistics analysis. Regression Poisson of all models was implemented in R using a generalized linear model function and robust variance control with sandwich package^30^.

## Supplementary Information

Supplementary Information 1.

Supplementary Information 2.

## Data Availability

The genomes of the studied isolates are loaded in GenBank with the accession numbers SAMN26037035-SAMN26037070 and the BioProject ID PRJNA808219, https://www.ncbi.nlm.nih.gov/bioproject/?term=PRJNA808219.

## References

[1] Esmail H, Barry CE, Young DB, Wilkinson RJ (2014). The ongoing challenge of latent tuberculosis. Philos. Trans. R. Soc. Lond. Ser. B, Biol. Sci..

[2] Getahun H, Matteelli A, Chaisson RE, Raviglione M (2015). Latent *Mycobacterium tuberculosis* infection. N Engl J Med..

[3] Veatch AV, Kaushal D (2018). Opening Pandora’s Box: Mechanisms of *Mycobacterium tuberculosis* Resuscitation. Trends Microbiol..

[4] Gibson SER, Harrison J, Cox JAG (2018). Modelling a silent epidemic: a review of the in vitro models of latent tuberculosis. Pathog (Basel, Switzerland)..

[5] Behr MA, Edelstein PH, Ramakrishnan L (2018). Revisiting the timetable of tuberculosis. BMJ.

[6] Weller C, Wu M (2015). A generation-time effect on the rate of molecular evolution in bacteria. Evolution.

[7] Hershkovitz I, Donoghue HD, Minnikin DE, Besra GS, Lee OY-C, Gernaey AM (2008). Detection and molecular characterization of 9,000-year-old *Mycobacterium tuberculosis* from a Neolithic settlement in the Eastern Mediterranean. PLoS ONE.

[8] Wirth T, Hildebrand F, Allix-Béguec C, Wölbeling F, Kubica T, Kremer K (2008). Origin, spread and demography of the *Mycobacterium tuberculosis* complex. PLoS Pathog..

[9] Arnold C (2007). Molecular evolution of *Mycobacterium tuberculosis*. Clin. Microbiol. Infect. Off. Publ. Eur. Soc. Clin. Microbiol. Infect Dis..

[10] Ford CB, Lin PL, Chase MR, Shah RR, Iartchouk O, Galagan J (2011). Use of whole genome sequencing to estimate the mutation rate of *Mycobacterium tuberculosis* during latent infection. Nat. Genet..

[11] Colangeli R, Arcus VL, Cursons RT, Ruthe A, Karalus N, Coley K (2014). Whole genome sequencing of *Mycobacterium tuberculosis* reveals slow growth and low mutation rates during latent infections in humans. PLoS ONE.

[12] Colangeli R, Gupta A, Vinhas SA, Chippada Venkata UD, Kim S, Grady C (2020). *Mycobacterium tuberculosis* progresses through two phases of latent infection in humans. Nat. Commun..

[13] Cacho J, Pérez Meixeira A, Cano I, Soria T, Ramos Martos A, Sánchez Concheiro M (2007). Recurrent tuberculosis from 1992 to 2004 in a metropolitan area. Eur. Respir. J..

[14] Caminero JA, Pena MJ, Campos-Herrero MI, Rodríguez JC, Afonso O, Martin C (2001). Exogenous reinfection with tuberculosis on a European island with a moderate incidence of disease. Am. J. Respir. Crit. Care Med..

[15] Afshar B, Carless J, Roche A, Balasegaram S, Anderson C (2019). Surveillance of tuberculosis (TB) cases attributable to relapse or reinfection in London, 2002–2015. PLoS ONE.

[16] Guerra-Assunção JA, Houben RMGJ, Crampin AC, Mzembe T, Mallard K, Coll F (2015). Recurrence due to relapse or reinfection with *Mycobacterium tuberculosis*: a whole-genome sequencing approach in a large, population-based cohort with a high HIV infection prevalence and active follow-up. J. Infect. Dis..

[17] Shanmugam S, Bachmann NL, Martinez E, Menon R, Narendran G, Narayanan S (2021). Whole genome sequencing based differentiation between re-infection and relapse in Indian patients with tuberculosis recurrence, with and without HIV co-infection. Int. J. Infect. Dis. IJID Off. Publ. Int. Soc. Infect. Dis..

[18] Lalor MK, Casali N, Walker TM, Anderson LF, Davidson JA, Ratna N (2018). The use of whole-genome sequencing in cluster investigation of a multidrug-resistant tuberculosis outbreak. Eur. Respir. J..

[19] Gagneux S (2018). Ecology and evolution of *Mycobacterium tuberculosis*. Nat. Rev. Microbiol..

[20] Moreno-Molina M, Shubladze N, Khurtsilava I, Avaliani Z, Bablishvili N, Torres-Puente M (2021). Genomic analyses of *Mycobacterium tuberculosis* from human lung resections reveal a high frequency of polyclonal infections. Nat. Commun..

[21] Xu Y, Liu F, Chen S, Wu J, Hu Y, Zhu B (2018). In vivo evolution of drug-resistant *Mycobacterium tuberculosis* in patients during long-term treatment. BMC Genomics.

[22] Pérez-Lago L, Monteserin J, Paul R, Maus SR, Yokobori N, Herranz M (2022). Recurrences of multidrug-resistant tuberculosis: Strains involved, within-host diversity, and fine-tuned allocation of reinfections. Transbound. Emerg. Dis..

[23] Gonzalo-Asensio J, Pérez I, Aguiló N, Uranga S, Picó A, Lampreave C (2018). New insights into the transposition mechanisms of IS6110 and its dynamic distribution between *Mycobacterium tuberculosis* Complex lineages. PLoS Genet..

[24] Tanaka MM (2004). Evidence for positive selection on *Mycobacterium tuberculosis* within patients. BMC Evol Biol..

[25] van Soolingen D, de Haas PE, Hermans PW, van Embden JD (1994). DNA fingerprinting of *Mycobacterium tuberculosis*. Methods Enzymol..

[26] Van Embden JDA, Cave MD, Crawford JT, Dale JW, Eisenach KD, Gicquel B (1993). Strain identification of *Mycobacterium tuberculosis* by DNA fingerprinting: Recommendations for a standardized methodology. J. Clin. Microbiol..

[27] Kamerbeek J, Schouls L, Kolk A, Van Agterveld M, Van Soolingen D, Kuijper S (1997). Simultaneous detection and strain differentiation of *Mycobacterium tuberculosis* for diagnosis and epidemiology. J. Clin. Microbiol..

[28] Robinson JT, Thorvaldsdóttir H, Winckler W, Guttman M, Lander ES, Getz G (2011). Integrative genome viewer. Nat. Biotechnol..

[29] Coll F, McNerney R, Guerra-Assunção JA, Glynn JR, Perdigão J, Viveiros M (2014). A robust SNP barcode for typing *Mycobacterium tuberculosis* complex strains. Nat. Commun..

[30] Zeileis, A., Köll, S., & Graham, N. Various versatile variances: an object-oriented implementation of clustered covariances in R.* J. Stat. Softw.* **95**, (1 SE-Articles), 1–36 (2020).

