# Author Correction: Estimation of the mutation rate of *Mycobacterium tuberculosis* in cases with recurrent tuberculosis using whole genome sequencing

**DOI:** 10.1038/s41598-023-30479-1

**Published:** 2023-02-27

**Authors:** Jessica Comín, Alberto Cebollada, Aragonese Working Group on Molecular Epidemiology of Tuberculosis (EPIMOLA), María José Iglesias, Daniel Ibarz, Jesús Viñuelas, Luis Torres, Juan Sahagún, María Carmen Lafoz, Felipe Esteban Juanas, María Carmen Malo, Sofía Samper

**Affiliations:** 1 Instituto Aragonés de Ciencias de la Salud, C/de San Juan Bosco, 13, 50009 Zaragoza, Spain; 2 Unidad de Biocomputación, Instituto Aragonés de Ciencias de la Salud, C/de San Juan Bosco, 13, 50009 Zaragoza, Spain; 3 Fundación IIS Aragón, C/de San Juan Bosco, 13, 50009 Zaragoza, Spain; 4 CIBER de Enfermedades Respiratorias, Av. Monforte de Lemos, 3-5. Pabellón 11, Planta 0, 28029 Madrid, Spain; 5 Universidad de Zaragoza, Zaragoza, Spain; 6 Hospital Universitario Miguel Servet, Zaragoza, Spain; 7 Hospital General Universitario San Jorge, Huesca, Spain; 8 Hospital Universitario Lozano Blesa, Zaragoza, Spain; 9 Salud Pública, Gobierno de Aragón, Zaragoza, Spain

Correction to: *Scientific Reports* https://doi.org/10.1038/s41598-022-21144-0, published online 06 October 2022

The original version of this Article contained errors, where the values in the columns ‘[1–2 years]’, ‘[2–14 years]’ and ‘*p* value’ in Table 1, and consequently in the Results and the Discussion section were incorrect due to an error in data assembly. The original Table 1 and accompanying legend appear below.

**Table 1:** Risk factors of the 18 selected cases.

	[1–2 years]	(2–14 years]	*p *value
	*N *= 6	*N *= 12	
Gender			0.066
Male	7 (87.5%)	4 (40.0%)	
Female	1 (12.5%)	6 (60.0%)	
HIV status:			0.041
Negative	2 (25.0%)	7 (87.5%)	
Positive	6 (75.0%)	1 (12.5%)	
Alcohol consumption:			1.000
High	1 (25.0%)	3 (42.9%)	
Low/No	3 (75.0%)	4 (57.1%)	
Immunosuppression:			0.072
No	1 (20.0%)	6 (85.7%)	
Yes	4 (80.0%)	1 (14.3%)	
Smoker:			0.576
No	2 (50.0%)	2 (28.6%)	
Yes	2 (50.0%)	5 (71.4%)	
Users of IV drugs:			0.491
No	2 (50.0%)	6 (85.7%)	
Yes	2 (50.0%)	1 (14.3%)	

As a result, in the Results section, under the subheading ‘Patient selection and risk factors’,

“HIV status was significant (*p* value = 0.041) between the two groups, showing that HIV positive patients suffered relapse in the first two years more frequently than HIV negative patients.”

now reads:

“HIV status was significant (*p* value = 0.035) between the two groups, showing that HIV positive patients suffered relapse in the first two years more frequently than HIV negative patients.”

Additionally, the following sentence was removed:

“The gender was marginally significant for males, suggesting a trend of males suffering relapses in the first two years more frequently than females (*p* value = 0.066).”

Furthermore, in the Discussion section,

“Regarding the epidemiological and risk factors of the relapsed TB cases studied, we found that relapse was significantly earlier in HIV positive patients (in the first two years since the first episode) when compared to HIV negative patients (*p* value = 0.041), what would be in accordance with a compromised immune system.”

now reads:

“Regarding the epidemiological and risk factors of the relapsed TB cases studied, we found that relapse was significantly earlier in HIV positive patients (in the first two years since the first episode) when compared to HIV negative patients (*p* value = 0.035), what would be in accordance with a compromised immune system.”

Finally, the following sentence was removed:

“We also found a trend that males suffered relapse earlier than females, which could be linked to other risk factors such as the use of IV drugs, smoking and the HIV status, which were more frequent in males in our study population.”

The original Article has been corrected.

